# General Characteristics, Biomedical and Dental Application, and Usage of Chitosan in the Treatment of Temporomandibular Joint Disorders: A Narrative Review

**DOI:** 10.3390/pharmaceutics14020305

**Published:** 2022-01-27

**Authors:** Marcin Derwich, Lukasz Lassmann, Katarzyna Machut, Agata Zoltowska, Elzbieta Pawlowska

**Affiliations:** 1 ORTODENT, Specialist Orthodontic Private Practice in Grudziadz, 86-300 Grudziadz, Poland; 2 Dental Sense, Dental Private Practice in Gdansk, 80-283 Gdansk, Poland; lassmann.lukas@gmail.com; 3 Department of Endodontic Dentistry, Medical University of Gdansk, 80-210 Gdansk, Poland; katarzyna.machut@gumed.edu.pl (K.M.); azolt@gumed.edu.pl (A.Z.); 4 Department of Orthodontics, Medical University of Lodz, 90-419 Lodz, Poland; elzbieta.pawlowska@umed.lodz.pl

**Keywords:** chitosan, hyaluronic acid, TMD, temporomandibular joint disorders, temporomandibular joints

## Abstract

The aim of this narrative review was to present research investigating chitosan, including its general characteristics, properties, and medical and dental applications, and finally to present the current state of knowledge regarding the efficacy of chitosan in the treatment of temporomandibular disorders (TMDs) based on the literature. The PICO approach was used for the literature search strategy. The PubMed database was analyzed with the following keywords: (“chitosan”[MeSH Terms] OR “chitosan”[All Fields] OR “chitosans”[All Fields] OR “chitosan s”[All Fields] OR “chitosane”[All Fields]) AND (“temporomandibular joint”[MeSH Terms] OR (“tem-poromandibular”[All Fields] AND “joint”[All Fields]) OR “temporomandibular joint”[All Fields] OR (“temporomandibular”[All Fields] AND “joints”[All Fields]) OR “temporo-mandibular joints”[All Fields]). After screening 8 results, 5 studies were included in this review. Chitosan presents many biological properties and therefore it can be widely used in several branches of medicine and dentistry. Chitosan promotes wound healing, helps to control bleeding, and is used in wound dressings, such as sutures and artificial skin. Apart from its antibacterial property, chitosan has many other properties, such as antifungal, mucoadhesive, anti-inflammatory, analgesic, antioxidant, antihyperglycemic, and antitumoral properties. Further clinical studies assessing the efficacy of chitosan in the treatment of TMD are required. According to only one clinical study, chitosan was effective in the treatment of TMD; however, better clinical results were obtained with platelet-rich plasma.

## 1. Introduction

Temporomandibular disorders (TMDs) is a broad term encompassing dysfunction of the temporomandibular joints (TMJs) and/or the masticatory musculature. The most important feature of TMD is pain, followed by restricted or limited jaw movement, and joint noises during jaw movement [1]. Pain-related TMD, including, among others, myalgia and arthralgia, and intra-articular TMD, including different types of disc displacements, degenerative joint disease, and subluxation [1], exist.

The overall prevalence of TMD was found to be approximately 31% for adults/elderly and 11% for children/adolescents, and the most common type of TMD is disc displacement with reduction [2]. Persistent and recurrent pain in the area of TMJs may lead to psychological discomfort, physical disability, and functional limitations, and therefore may reduce oral health-related quality of life [3,4,5,6].

**Figure 1:**
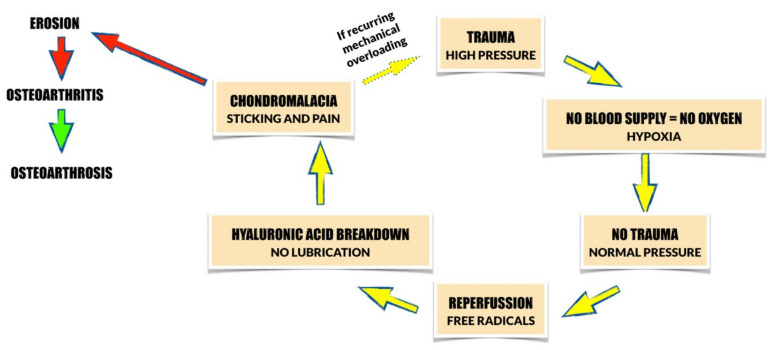
Schematic changes that occur within TMJs due to mechanical overloading based on the literature [8]. Yellow arrow: reversible progression; red arrow: irreversible progression; green arrow: remission.

The etiology of TMD is multifactorial [7]. One of the most important potential causes of TMD is mechanical overloading. Excessive intraarticular forces may cause temporary hypoxia in vascular-supplied structures. When the load is reduced and the intraarticular pressure decreases, blood is reperfused into the capillaries that supply the joint structures. Repeatable cycles of hypoxia and reperfusion lead to the release of free radicals into the synovial fluid. Free radicals have been found to inhibit biosynthesis of hyaluronic acid, and break down already existing chains of hyaluronic acid. Hyaluronic acid protects the phospholipids that line the surfaces of the joints and provide important hydration [8,9,10,11,12,13,14,15,16,17]. Without phospholipids, articular surfaces wear off, which leads to TMJs’ destruction and so-called “sticking” [18,19,20]. Afterwards, non-inflammatory osseocartilaginous pathology, known as chondromalacia, develops [21,22]. Chondromalacia is reversible in its early stages. However, if the mechanical loading exceeds the capacity of the articular tissues, irreversible changes occur within the TMJs. This may lead to sticking of the articular surfaces (adherences), and eventually to disc displacements [22,23]. Figure 1 presents the schematic changes that occur within the TMJs due to mechanical overloading based on the literature [8].

Different treatment modalities for TMD have been discussed, including conservative methods of treatment (physiotherapy, occlusal splint therapy, pharmacotherapy), less invasive surgical procedures (arthrocentesis, intraarticular injections), and invasive surgical procedures (arthroscopy, open joint surgery) [24,25]. The choice of an adequate treatment option depends on the initial diagnosis, history of previous treatment, and intensity of reported symptoms [24,25].

Minimally invasive surgical procedures, including arthrocentesis and intraarticular injections, are very effective in TMJs’ pain reduction and therefore can be recommended either additionally to conservative therapy or even as a first-line therapy [25]. Arthrocentesis can be performed either alone or in combination with intraarticular injections. So far, intraarticular injections of hyaluronic acid (HA), corticosteroids (CS), and platelet-rich plasma (PRP) have been examined. However, the results of intraarticular injections performed after arthrocentesis were not superior to the results obtained with arthrocentesis performed alone. In case of intraarticular injections performed without previous arthrocentesis, better results were obtained with HA compared to either CS or physiologic saline solution [26].

Although a lot is known about the use of HA, CS, and PRP in the treatment of TMD, none of these substances are considered to unquestionably be the gold standard. Contemporary medicine is looking for new solutions and new biomaterials for tissue regeneration. One of the very interesting materials in terms of tissue regeneration is chitosan, a deacetylated form of chitin, a linear semi-crystalline polymer and the most naturally abundant polysaccharide after cellulose [27,28]. Nowadays, chitosan is one bone tissue engineering scaffold. It has a similar chemical structure to glycosaminoglycans (the main components of connective tissue extracellular matrix, ECM), and it can be shaped to specific bone defects and fabricated as membranes, fibers, nanoparticles, hydrogels, or through 3D printing [29,30,31]. Moreover, chitosan has been found to be effective in the process of cartilage regeneration, and in relieving osteoarthritis [32,33,34,35]. Therefore, chitosan may be an effective agent in the treatment of TMD.

The aim of this narrative review was to present research investigating chitosan, including its general characteristics, properties, and medical and dental applications, and finally to present the current state of knowledge regarding the efficacy of chitosan in the treatment of TMD based on the literature.

## 2. Methodology of the Literature Search Strategy

### 2.1. Clinical Question

What is the efficacy of chitosan in the treatment of TMD in humans and animals based on the literature?

### 2.2. Inclusion and Exclusion Criteria

Table 1 presents the inclusion and exclusion criteria used for the narrative review.

### 2.3. The PICO Approach

We used the PICO approach to properly develop literature search strategies for this review:

Population:

Patients who were diagnosed with TMD, and animal models.

Intervention:

Intraarticular injections of chitosan in patients diagnosed with TMD, usage of chitosan-based scaffolds placed in TMJs

Comparison:

Intraarticular injection of other substances, including: PRP, HA, CS, arthrocentesis or arthroscopy alone, open joint surgeries, and placebo. Randomized controlled trials (RCTs), randomized clinical trials, case-control studies, case reports, and animal studies were included in the review.

Outcome:

Increased maximum mouth opening and decreased pain in the temporomandibular joint area.

### 2.4. Search Strategy

The PubMed database was analyzed with the following keywords: (“chitosan”[MeSH Terms] OR “chitosan”[All Fields] OR “chitosans”[All Fields] OR “chitosan s”[All Fields] OR “chitosane”[All Fields]) AND (“temporomandibular joint”[MeSH Terms] OR (“temporomandibular”[All Fields] AND “joint”[All Fields]) OR “temporomandibular joint”[All Fields] OR (“temporomandibular”[All Fields] AND “joints”[All Fields]) OR “temporomandibular joints”[All Fields]). After screening 8 results, 5 studies were included in this review.

**Figure 2:**
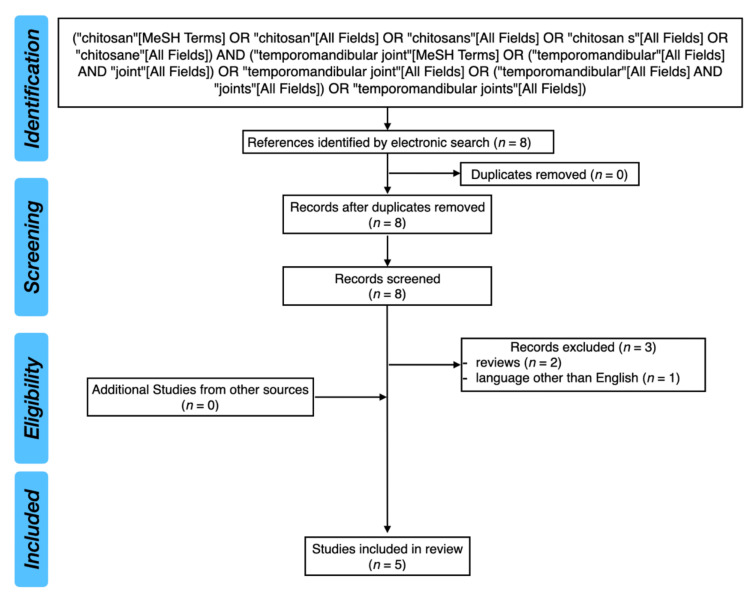
PRISMA flow diagram for the review of the literature.

Figure 2 presents the PRISMA flow diagram for the review of the literature.

### 2.5. Cohen’s Kappa Coefficient

Cohen’s kappa coefficient between the reviewers was 1.00.

## 3. Chitosan: General Characteristics

Chitin (β-(1–4)-poly-N-acetyl-D-glucosamine) is a natural mucopolysaccharide with a structure similar to cellulose [36,37] and is one of the most common biopolymers. Chitin is a compound found in fungi cell walls and forms outer-shell structures, such as the carapaces of marine invertebrates, e.g., crabs, lobsters, and shrimps, or the exoskeletons of insects and arthropods, and endoskeletons of mollusks [29,36,37].

The first study and isolation of chitin from fungal species was made by a French chemist Henri Braconnot in 1811. Chitin is synthesized from uridine diphosphate N-acetylglucosamine (UDP-N-acetylglucosamine) by the chitin synthase [29,38]. Chitin has a limited range of applications as scaffolds to support tissue regeneration due to its insolubility [39,40]. A water-soluble derivate of chitin, chitosan, is more useful in multiple fields as it is biocompatible, non-toxic, low allergenic, low immunogenic, and biodegradable [36,40]. Chitosan has been found to be an interesting material in many fields, including medicine, agriculture, food processing, nutritional enhancement, cosmetics, and waste and water treatment [41].

**Figure 3:**
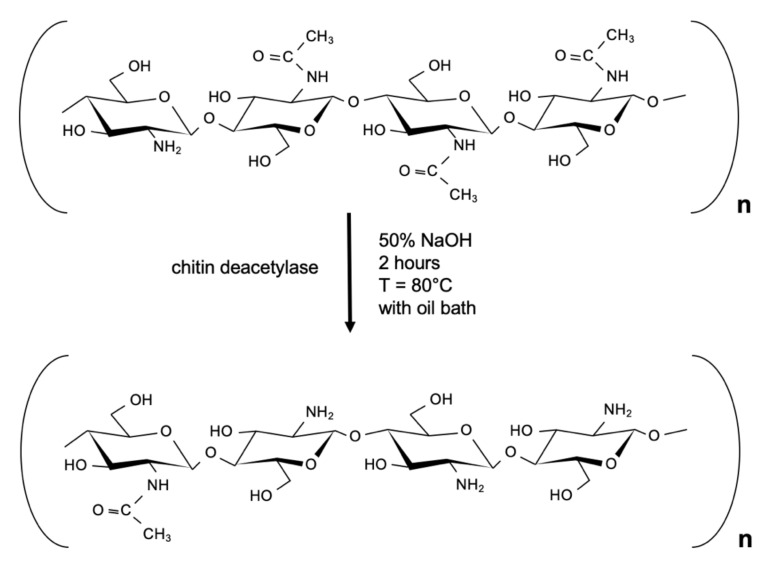
The reaction of chitin deacetylation with chitin deacetylase.

Chitosan is a polycationic linear polysaccharide composed of varying amounts of deacetylated β-(1–4)glucosamine (GlcN) and acetylated N-acetyl-glucosamine (GlcNAc) residues [42,43]. It is obtained from chitin in a deacetylation process with chitin deacetylase (of bacterial or fungal origin) or in an alkaline hydrolysis [40,44,45]. Figure 3 presents the reaction of chitin deacetylation.

Chitosan was obtained for the first time by treating chitin with potassium hydroxide in the second half of the 19th century, but the chemical structure of chitosan was announced in 1950, and the name “chitosan” was proposed by Hoppe-Seyler in 1984 [36,46,47]. Fungi belonging to the classes Zygomycetes and Mucorales are capable of producing chitosan, as a natural structural element of their cell walls [47]. This natural source of chitosan is under further research for use in tissue engineering [47].

The main sources of chitosan are the carapaces of shrimps due to their small thickness. The shells are cleaned, dried, and smashed into smaller pieces. The typical protocol is divided into four steps: demineralization, deproteinization, decolorization, and deacetylation. All processes can be chemical or biological (by enzymatic proteolysis or bacterial fermentation). For biomedical use, the final product has to be purified [48].

The final reaction of chitin deacetylation is usually incomplete and chitosan contains acetamide groups. The presence of an amine group on each deacetylated unit in weak acidic solution (H^+^) can be positively charged (NH3^+^). The positive electric energy on the surface of chitosan in an acidic environment makes it possible to form complexes with other synthetic or natural polymers, and also makes chitosan soluble. These physicochemical properties ensure it biocompatibility and bacteriostasis and the promotion of cell growth [49,50]. Chitosan can be enzymatically degraded by the family of enzymes named chitosanases. Chitosanases are glycosyl hydrolases that break β-1,4-glycosidic bonds [51].

The positive electric charge density depends on the degree of deacetylation (% DD), which is described by the percentage of the molar fraction of deacetylated units, environmental pH, and molecular weights, which range from 300 to more than 1000 kilodaltons [42,52]. Chitosan oligomers are soluble over a wide pH range, but chitosan with a higher molecular weight chitosan forms with a higher % DD are only soluble in acidic solutions [29,30,50].

Chitosan can bind with cholesterol, triglycerides, proteins, and metal ions, so it is commonly used as a chelating agent. Moreover, the cationic chitosan can easily bind negatively charged molecules, such as plasmid DNA. The DNA-chitosan nanoparticles (DNA-CS NP) are small-sized complexes, ranging from 20 to 500 nm, and can be transported into the cell through endocytosis or pinocytosis, and are stable during oral delivery. DNA-CS has been used in gene delivery therapy in orthopedic bone regeneration [53,54,55,56].

Chitosan is able to promote the accumulation of anionic platelets and erythrocytes, so it is useful for controlling bleeding and is used in wound dressings, such as sutures and artificial skin, which has been approved by the Food and Drug Administration (FDA). The antibacterial property of chitosan is also useful in wound healing, due to the ability to form electrostatic interactions between chitosan’s positive charge groups and anions presented on the cell wall (bacterial growth inhibition). This effect lasts up to 7 days [57].

Apart from its antibacterial property, chitosan has many others, such as antifungal, mucoadhesive, anti-inflammatory, analgesic, antioxidant, antihyperglycemic, and antitumoral properties. All the aforementioned abilities are used in medicine, for example, in periodontology, guided tissue engineering, dermatology, and pharmacology (in drug delivery systems) [53,54,58,59].

## 4. Chitosan in Comparison to Hyaluronic Acid

Chitosan and hyaluronic acid (HA) are polysaccharides with similar biochemical structures [60]. However, some differences between these molecules exist and therefore it seems rational to compare their structures and properties.

**Figure 4:**
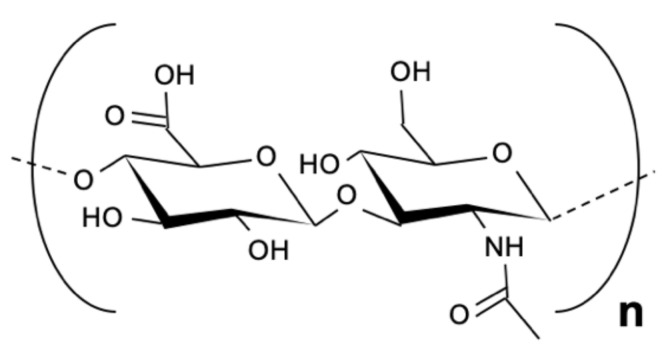
The skeletal structure of hyaluronic acid.

HA, also known as hyaluronan, is a naturally occurring non-sulfated mucopolysaccharide in human connective, epithelial, and neural tissues [61,62]. HA is part of the glycosaminoglycans (GAG) family and it is the only mucopolysaccharide that is synthesized on the inner surface of the cell membrane rather than in the Golgi apparatus. HA is synthesized by HA synthases. The HA molecule is anionic and it is composed of repeating units of disaccharides: D-glucuronic acid and N-acetylglucosamine, which are bonded by β-(1–4) and β-(1–3)glycosides linkages [61,62,63]. Figure 4 presents the skeletal structure of HA.

HA presents significant water solubility and hydrophilicity, due to its ability to bind water molecules [64].

In some stress conditions, such as injury, high molecular weight HA (HMWHA) can be fragmented into smaller chains, known as low molecular weight HA (LMWHA). Probably, LMWHA is a proinflammatory molecule, in contrast to HMWHA, which has well-documented anti-inflammatory and immunosuppressive properties [65].

The catabolism of HA chains can be either enzymatic with hyaluronidases from the hydrolysis family, or chemical. There are three enzymatic isoforms of hyaluronidase: hyaluronidase-1 (HYAL-1), hyaluronidase-2 (HYAL-2), and hyaluronidase-3 (HYAL-3). The chemical degradation is caused by reactive oxygen forms. These free-radicals of HA degradation can be prevented by superoxide dismutase [63,66].

Existing studies have indicated that HA has good biocompatibility and a broad distribution of HA receptors within the whole body. These abilities have been successfully applied in many branches of medicine. HA derivates are commonly used in orthopedics as a joint lubricant, osteoarthritis or rheumatoid arthritis therapy, aesthetic dermatology as dermal filler, ophthalmology in corneal wound healing, or the treatment of dry eye syndrome. HA is widely used in drug delivery systems [63,66,67,68]. HA can also be used in dentistry to help in periodontal tissue healing, as a topical gel in gingivitis and chronic periodontitis treatment, and in reducing postoperative pain after surgical procedures (such as implant and sinus lift). Moreover, HA shows osteoinductive properties, which are used during the regeneration of bone defects. HA is recommended in the therapy of mouth dryness, for example, in Sjorgen’s syndrome [69,70].

Table 2 presents a comparison of the physical and biochemical properties of chitosan and HA based on the literature.

## 5. Properties of Chitosan and its Biomedical Application

The antimicrobial activity of chitosan was first described by Allan and Hadwiger [74]. Two potential antimicrobial mechanisms have been identified depending on chitosan’s molecular weight. Low molecular weight (LMW) chitosan has been found to penetrate bacterial walls, bind with DNA, and control gene expression. Contrary to this, high molecular weight (HMW) chitosan binds with negatively charged components on the bacterial cell wall, forming a polymer membrane that prevents nutrients from entering the cell [75,76].

The process of wound healing comprises five overlapping stages, namely: homeostasis, inflammation, migration, proliferation, and maturation [77]. Immediately after the application of chitosan to a fresh wound, the immune system is activated, the number of cytokines released from thrombocytes increases, and formation of a blood clot is accelerated. Next, leukocyte phagocytosis is caused by vascular endothelial cell activity and granulocyte migration. Macrophages turn into giant cells that break down large amounts of collagen, which prevents scarring. As a result of the contact between migrating leukocytes and chitosan, physiologically active substances are released, which consequently intensifies the activity of monocytes, macrophages, vascular endothelial cells, and fibroblasts. The cascade of these reactions promotes the formation of granulation tissue and stimulates angiogenesis [78].

Chitosan also has the ability to inhibit matrix metalloproteinases (MMPs), enzymes capable of degrading components of the extracellular matrix, including collagens, laminin, and fibronectin [79]. MMPs play a significant role in processes related to tissue remodeling, including wound healing, arthritis, and neoplastic changes, significantly impeding regenerative processes. The inhibitory effect of chitosan on MMPs has been explained as the interaction between two mechanisms: inhibition of MMPs’ expression at the transcriptional level, and limitation of the access to zinc ions, which are considered to be cofactors of MMPs [80,81]. Shikhman et al. [82] showed that the chondroprotective efficacy of GlcNAc was better than viscosupplementation treatment with hyaluronan.

**Figure 5:**
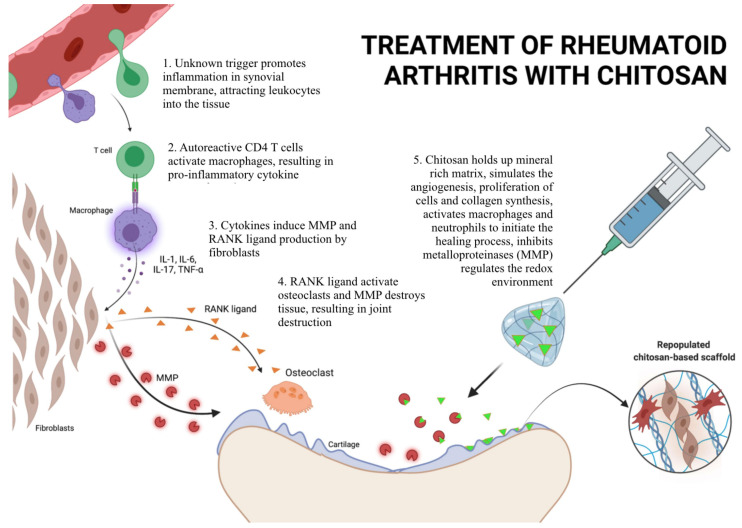
Treatment of rheumatoid arthritis with chitosan based on the literature [83]. IL-1: interleukin 1; IL-6: interleukin 6; IL-17: interleukin 17; MMP: matrix metalloproteinases; RANK ligand: Receptor Activator for Nuclear Factor κ B Ligand; T cell: lymphocyte type T; TNFα: tumor necrosis factor α. Figure 5 was created with BioRender.com. (accessed on 17 December 2021.).

Figure 5 presents the exemplary mechanism of treatment of rheumatoid arthritis (RA) with chitosan. Inflammation of the synovial membrane within the joint is initiated by an unknown trigger. Autoreactive lymphocytes and macrophages are attracted to the inflamed tissue. Next, autoreactive CD4 T cells activate macrophages, which results in the production of proinflammatory cytokines. Proinflammatory cytokines activate fibroblasts to produce MMPs. The tumor necrosis factor (TNF) family cytokine receptor activator for nuclear factor κ B ligand (RANK ligand) is the primary activator of bone-destroying osteoclasts. Intraarticular injection of chitosan promotes cartilage formation, inhibits MMPs, regulates the redox environment, stimulates angiogenesis, and initiates the healing processes [83].

Articular cartilage presents limited repair capacity [84]. The structure of chitosan is similar to the structure of the repeating polysaccharide units in articular cartilage [85,86], making the characteristics of chitosan similar to those of hyaluronic acid and GAG in the ECM [87]. These GAG-analogous structures are also involved in the synthesis of chondroitin sulfate, HA, and collagen type II [88]. Therefore, chitosan, especially in the form of a hydrogel [89], can simulate the ECM of articular cartilage and promote cartilage formation as a natural scaffold for repairing cartilage defects [90]. These unique properties of the chitosan scaffold are related to the size and orientation of the chitosan pores [85]. The properties of chitosan in wound healing also include the ability to stimulate the production of fibroblasts by the fibroblast growth factor [91]. To sum up, the justification for using chitosan in bone tissue engineering is its ability to maintain mineral-rich matrix deposition, and its osteoconduction, biocompatibility, and biodegradability [52].

Moreover, many studies have shown that chitosan and its derivatives also present antitumor activity [92], which has been found to be related to the increase in lymphokine production resulting in the proliferation of cytolytic T cells [93]. It is supposed that the increased secretion of IL-1 and IL-2 may cause an anti-tumor effect through the maturation and infiltration of cytolytic T lymphocytes [94]. However, other studies have shown that chitosan may also directly affect cancer cells and may inhibit tumor cell proliferation by caspase-3 induced apoptosis [95,96].

**Figure 6:**
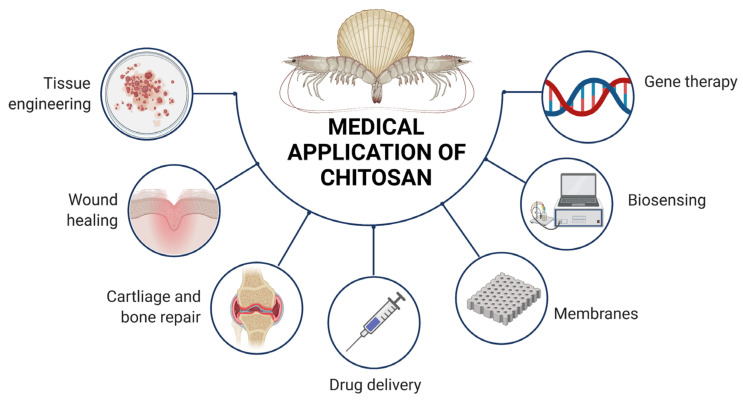
Medical application of chitosan based on the literature [74,75,76,77,78,79,80,81,82,83,84,85,86,87,88,89,90,91,92,93,94,95,96,97,98,99,100,101]. Figure 6 was created with BioRender.com. (accessed on 17 December 2021.).

Figure 6 summarizes the medical application of chitosan based on the literature [74,75,76,77,78,79,80,81,82,83,84,85,86,87,88,89,90,91,92,93,94,95,96,97,98,99,100,101].

## 6. Chitosan Application in Dentistry

**Figure 7:**
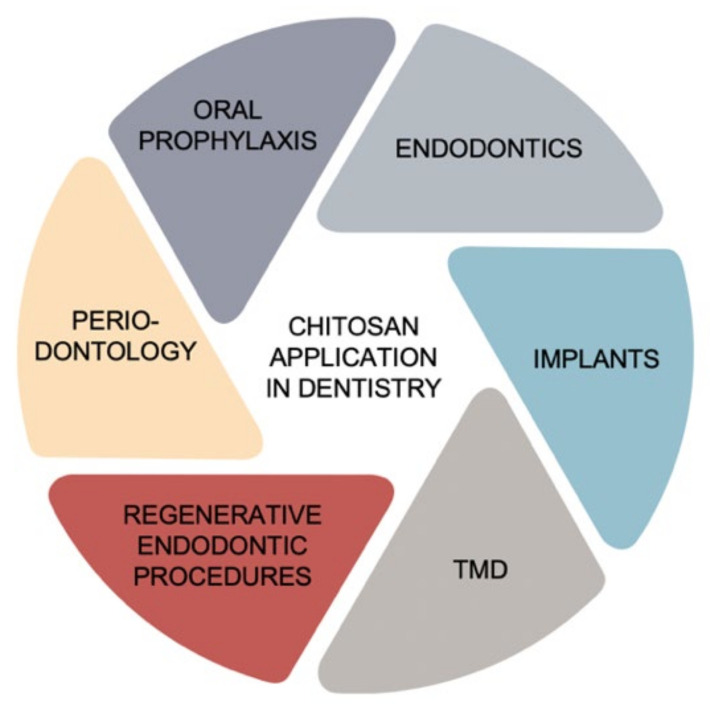
Application of chitosan in dentistry based on the literature [102,103,104,105,106,107,108,109,110,111,112,113,114,115,116]. TMDs: temporomandibular joint disorders.

Figure 7 schematically presents the application of chitosan in dentistry based on the literature [102,103,104,105,106,107,108,109,110,111,112,113,114,115,116].

### 6.1. Oral Prophylaxis

Chitosan’s cationic groups interact with negative-charge components of bacteria’s cell surface. Chitosan is known to have better inhibitory (bactericidal and bacteriostatic) effects on Gram-negative than Gram-positive bacteria [102]. Moreover, chitosan’s amino groups (NH3^+^) may neutralize the acidic environment within dental plaque and therefore chitosan is able to maintain the pH of plaque above the critical value required for enamel demineralization [103].

Costa et al. [104] found that chitosan-based mouthwash could inhibit microbial adhesion (Enterococcus faecalis, S. mutans, Candida albicans, and Prevotella intermedia), and biofilm formation while at the same time promoting the dissolution of already formed bacterial colonies. These results suggest that chitosan-based mouthwash can be used for prophylaxis of dental caries, periodontitis, and candidiasis.

Finally, chitosan has been found to interact with hydroxyapatite on the tooth surface. This property of chitosan, especially with nanoparticles, can inhibit biofilm formation on enamel and dentin, and indirectly prevent tooth demineralization [105]. In addition to this, chitosan is also able to penetrate the enamel and rebuild demineralized enamel and prevent the progression of demineralization [106].

### 6.2. Endodontics

Root canal treatment (RCT) is performed to remove inflamed or necrotic pulp infected by bacteria and to chemo-mechanically debride the root canal system [107]. Several reports have been published regarding the antibacterial activity of chitosan with nanoparticles (CSNPs) against pathogenic species, including S. mutans, E. faecalis, and P. gingivalis. Due to their potential benefits, CSNPs could be added to calcium hydroxide for temporary root canal filling or to endodontic sealers [106,108].

Chitosan has also been used in regenerative endodontic procedures [109]. Chitosan-based porous scaffolds can be enriched with growth factors and bioactive molecules. Released signaling biomolecules increase the expression of odontoblastic markers, such as dentin sialophosphoprotein, dentin matrix acidic phosphoprotein, and alkaline phosphate, which induce proliferation of dental pulp steam cells (DPSCs) and differentiation into odontoblasts [106]. Innovative chitosan-based scaffolds containing the bioactive molecules bone morphogenetic proteins (BMPs) and transforming growth factor-β1 (TGF-β1) have been found to promote cell adhesion, differentiation and proliferation of DPSCs, and odontoblast-like cells [106,110].

### 6.3. Periodontology

Chitosan can be used as a scaffold for the regeneration of periodontal tissues (in the treatment of periodontal pockets), because of its good biocompatibility, degradability by naturally occurring enzymes, appropriate physicochemical properties, and optimal molecule size. Injectable hydrogels with chitosan membranes can potentially be applied in drug delivery systems or in tissue engineering [106,111,112,113,114].

Moreover, chitosan scaffolds can be seeded by multi-potent dental mesenchymal stem cells, such as stem cells from human exfoliated deciduous teeth (SHED) and human periodontal ligament cells (HPLCs). This application of chitosan can be successfully used in periodontal tissue regeneration by activating cementoblasts and osteoblasts to form new tissue [115,116].

### 6.4. Implantology

Modifications of implant surfaces with chitosan are still being examined in terms of healing processes and osseointegration [98]. The degree of deacetylation (DDA%) may be crucial for improvement of the osteoinductive properties of chitosan coatings. Alnufaiy et al. [117] noticed that an increase in the DDA of chitosan coatings stimulated biomineralization and the formation of new osteoblasts.

## 7. Chitosan in the Treatment of TMD

Only three studies (one human and two animal studies) analyzing the efficacy of chitosan in the treatment of TMD were found.

Li et al. [118] retrospectively analyzed a group of 27 patients diagnosed with TMJ OA. In total, 15 patients (13 female and 2 male) were treated with 3 intraarticular injections of 1.0 mL of chitosan (1 intraarticular injection once per month). The remaining 12 patients (11 female and 1 male) were treated with 3 intraarticular injections of 1.0 mL of PRP. The authors analyzed the maximal interincisal opening (MIO), pain intensity, and TMJ sounds before the onset of the treatment, and 3 and 6 months after the end of the treatment. Li et al. [1] noticed that although MIO improved significantly in both groups during the observation period, significantly better results were obtained in the PRP group. However, it should also be emphasized that the average value of MIO before the onset of the treatment differed between the examined groups and was larger in patients from the PRP group. Pain intensity decreased in both groups throughout the observation period. However, significantly lower pain intensity at 6 months after the treatment was observed in patients treated with PRP compared to the chitosan group. Both groups reported relief of TMJ sounds. There were no statistically significant differences between the groups regarding the relief of TMJ sounds nor regarding the condylar bone reconstruction after the end of the treatment. Finally, complications after the intraarticular injections were observed only in the PRP group, including pain and swelling in the area of TMJ, and changes in the occlusion. All complications were transient.

Talaat et al. [119] performed an animal study in which they assessed the efficacy of thermosensitive hydrogels based on chitosan in terms of intraarticular controlled release of drugs in the rabbit temporomandibular joint. In total, 13 adult male rabbits were included into the study. The authors injected 0.2 mL of chitosan/β-glycerophosphate/hyaluronic acid into the rabbits’ left TMJs and 0.2 mL of control solution of hyaluronic acid into the rabbits’ right TMJs. Talaat et al. observed that the mean percentage of retention of hyaluronic acid was significantly higher within the TMJs injected with chitosan-based hydrogels compared to the controls (35.61 ± 6.68 vs. 12.13 ± 1.85, *p* < 0.001). According to the studies on the rabbit model, chitosan-based thermosensitive hydrogel appears to be an efficient controlled drug release system to the TMJ.

Li et al. [120] analyzed the effects of chitosan membrane on postsurgical TMJs’ intraarticular adhesions in goats with released retrodiscal tissues and therefore with completely displaced TMJ articular discs. The authors examined six goats. All of the goats underwent open joint surgeries, during which the retrodiscal tissues were cut off. Two of the goats served as a control, with no other additional surgeries or treatments performed. Four goats form the experimental group received a chitosan membrane during surgery, which was placed within the inferior joint space. According to the authors, chitosan membranes appeared to act as a barrier preventing adhesions, but the chitosan membranes also protected the condyles from damage. Moreover, the experimental group presented smooth condylar surfaces, with no hyperemia or adhesion, and significantly greater maximum passive mouth opening compared to the control group. The control group was diagnosed with severe intraarticular adhesions.

Table 3 presents the effectiveness of chitosan used in the treatment of TMD from in vivo studies [118,119,120].

Two studies analyzing the use of chitosan-based scaffolds in terms of tissue engineering in the treatment of TMD have also been published.

Bousnaki et al. [121] noticed that chitosan/alginate scaffolds stimulated the attachment and proliferation of dental pulp stem cells (DPSCs) and human nucleus pulposus cells (hNPCs). Moreover, DPSCs, which were cultured in chitosan/alginate scaffolds, presented increased expression of fibrocartilaginous markers. Therefore, the chitosan/alginate scaffolds were expected to be useful in regeneration in TMJ disc tissue by producing fibrocartilage tissue.

Tissue engineering using chitosan for the treatment of TMJ disorders was also analyzed by Wu et al. [122]. The authors found that synovium-derived mesenchymal stem cells (SDSCs) seeded in a fibrin/chitosan scaffold synthesized a fibrocartilage extracellular matrix. Wu et al. [122] indicated that above-described regenerative properties of SDSCs seeded in a fibrin/chitosan scaffold could be used for repairing TMJ articular disc perforation. The authors also emphasized that the cell seeding efficiency was significantly improved by using fibrin gel. There were significantly more SDSCs seeded in the fibrin/chitosan scaffold compared to the pure chitosan scaffold. However, the authors decided to combine fibrin gel with a porous chitosan scaffold to increase both the cell differentiation and synthesis of the extracellular matrix, and to support the fixation of the scaffold within the site of the disc defect using the adhesive properties of fibrin gel.

## 8. Conclusions

Chitosan presents many biological properties and therefore it can be widely used in several branches of medicine and dentistry. One of the most interesting properties of chitosan is its ability to promote wound healing, which makes chitosan useful in bone tissue engineering. There are currently inadequate numbers of clinical studies to assess the efficacy of chitosan for the treatment of TMD. According to only one clinical study, chitosan was effective in the treatment of TMD; however, better clinical results were obtained with platelet-rich plasma. Future perspectives should encompass assessment of the clinical efficacy of chitosan in the treatment of TMD and therefore further randomized, double-blind, and long-term clinical studies should be performed.

## Figures and Tables

**Table 1:** Inclusion and exclusion criteria used for the narrative review.

Criteria	List of Specific Criteria
Inclusion criteria	-Randomized controlled trials -Randomized clinical trials -Case-control studies -Case reports -Animal studies -Study population: humans diagnosed with TMD, animal models -Methods of treatment: intraarticular injections of chitosan, usage of chitosan-based scaffolds placed in the TMJs -Papers written in English
Exclusion criteria	-Comments -Systematic reviews and metanalyses -Study population: patients without the diagnosis of TMD -Methods of treatment: conservative methods of treatment (including physiotherapy, occlusal splint therapy and pharmacotherapy) -Papers written in languages other than English

TMD: temporomandibular joint disorder; TMJ: temporomandibular joint.

**Table 2:** Comparison of the physical and biochemical properties of chitosan and HA based on the literature.

Comparable Characteristic [References]	Chitosan	Hyaluronic Acid
Chemical structure [36,37,61,62]	mucopolysaccharide; deacetylated glucosamine (GlcN) and acetylated N-acetyl-glucosamine (GlcNAc)	mucopolysaccharide; D-glucuronic acid (D-GlcA) and N-acetylglucosamine (GlcNAc)
Electric charge [42,62]	cationic	anionic
Glycoside linkages [36,37,42,61,62,63,64,66]	β-(1–4)	β-(1–4) and β-(1–3)
Origin [29,36,37,61,62,71]	chitin ex. shells of shrimp or other crustaceans	1. ECM of vertebrate’s tissue, 2. bacterial sources, 3. chemoenzymatic sources
Appearance [44,64]	white or gray, translucent flake or powder solid, tasteless, odorless, non-toxic	transparent, viscous fluid or white powder
Molecular weight [42,64]	300–1000 kDa	4–20,000 kDa
Degradation enzyme [51,63,66]	chitosanases (hydrolasis)	hyaluronidases (hydrolasis)
Solubility [29,50,64,71]	only in solutions of pH ≤ 7	good in organic and inorganic solutions; HA depolimerizes when 4 < pH < 11
Physiochemical properties [36,37,40,42,61,62,63,66,71]	depend on molecular weight and acetylation degree: 1. bioadhesive, 2. biocompatible, 3. biodegradable	1. viscosity, 2. elasticity, 3. lubrication, 4. a high capacity for holding water, 5. biocompatible, 6. biodegradable, 7. bioadhesive
Biological properties [37,57,59,72,73]	1. mucoadhesive, 2. bactericidal, 3. fungicidal activity, 4. wound healing potential 5. antioxidant activity, 6. cholesterol and triglyceride trapping, 7. hypoglycemic effects	depend on the molecular size of HA: 1. regulation of cell division, migration, differentiation; 2. HA is an extracellular transmitting molecule in signaling pathways; 3. participates in tissue regeneration and inflammation 4. provides structural framework for cells

ECM: extracellular matrix; HA: hyaluronic acid.

**Table 3:** Effectiveness of chitosan used in the treatment of the TMD from the in vivo studies [118,119,120].

Reference	Study Design	Participants and Intervention	Endpoint and Results
Li et al. [118]	Retrospective, case control study	27 patients (24 women, 3 men, aged 25.74 ± 9.75): - chitosan group (15 patients): 3 intraarticular injections of 1.0 mL of chitosan once a month for 3 months - PRP group (12 patients): 3 intraarticular injections of 1.0 mL of PRP once a month for 3 months	Endpoint: 6 months MIO improved significantly in both groups, significantly better results presented PRP group. Pain intensity decreased in both groups, significantly lower pain intensity at 6 months after the treatment presented PRP group. Both of the groups reported relief of TMJ sounds. Complications after intraarticular injections were observed only in the PRP group.
Talaat et al. [119]	Animal study	13 adult male New Zealand white rabbits (18 weeks old on average; mean weight of 2.5 kg) - study group (left TMJs) injection of 0.2 mL of Chitosan/β-glycerophosphate/Hyaluronic Acid into rabbits’ left TMJs - control group (right TMJs) injection of 0.2 mL of Hyaluronic Acid (10 mg/mL)	Endpoint: 7 days Hydrogel scaffolds were able to retain significantly more of injected HA in the rabbits TMJs after 7 days compared to the control.
Li et al. [120]	Animal study	6 healthy adult goats: - control group (2 goats)—retrodiscal tissues were cut off without any other procedures - experimental group (4 goats)—retrodiscal tissues were cut off and chitosan membrane was placed between the articular disc and the condyle	Endpoint: 6 months Experimental group presented smooth condylar surfaces, with no hyperemia or adhesion, and significantly greater maximum passive mouth opening comparing to control group. Control group was diagnosed with severe intraarticular adhesions.

HA: hyaluronic acid; MIO: maximal interincisal opening; PRP: platelet-rich plasma; TMD: temporomandibular joint disorder; TMJ: temporomandibular joint.

## Data Availability

All data are contained within the article.
